# Molybdesum selenide-based platelet-rich plasma containing carboxymethyl chitosan/polyvinyl pyrrolidone composite antioxidant hydrogels dressing promotes the wound healing

**DOI:** 10.1186/s12951-024-02490-9

**Published:** 2024-05-09

**Authors:** Xiaoyi Zheng, Yongliang Ouyang, Hengwei Fan, Liying Zhang, Shige Wang, Yanbo Zeng, Lianghao Hu, Jiulong Zhao

**Affiliations:** 1 Department of Gastroenterology, Changhai Hospital, Naval Medical University, No. 168 Changhai Road, Shanghai, 200433 P. R. China; 2 School of Materials and Chemistry, University of Shanghai for Science and Technology, No. 516 Jungong Road, Shanghai, 200093 P. R. China; 3 Department of Hepatic Surgery Department, the Eastern Hepatobiliary Surgery Hospital, Navy Medical University, No. 225 Changhai Road, Shanghai, 200438 P. R. China

**Keywords:** Molybdenum selenide, Hydrogels, Oxygen radicals, Wound dressing

## Abstract

Excess free radicals at the wound site can cause an inflammatory response, which is not conducive to wound healing. Hydrogels with antioxidant properties can prevent inflammatory storms by scavenging free radicals from the wound site and inhibiting the release of inflammatory factors. In this study, we prepared the carboxymethyl chitosan (CMCS)/polyvinyl pyrrolidone (PVP)/Molybdenum (IV) Selenide (MoSe_2_), and platelet-rich plasma (PRP) (CMCS/PVP/MoSe_2_/PRP) hydrogels for accelerating the repair of wounds. In the hydrogels, the MoSe_2_ can scavenge various free radicals to reduce oxidative stress at the site of inflammation, endowed the hydrogels with antioxidant properties. Interestingly, growth factors released by PRP assisted the tissue repair by promoting the formation of new capillaries. CMCS as a backbone not only showed good biocompatibility and biodegradability but also played a significant role in maintaining the sustained release of growth factors. In addition, incorporating PVP enhanced the tissue adhesion and mechanical properties. The multifunctional composite antioxidant hydrogels have good swelling properties and biodegradability, which is completely degraded within 28 days. Thus, the antioxidant CMCS/PVP/MoSe_2_/PRP hydrogels provide a new idea for designing ideal multifunctional wound dressings.

**Supplementary Information:**

The online version contains supplementary material available at 10.1186/s12951-024-02490-9.

## Introduction

The skin plays an essential role in maintaining the homeostasis of the body and protecting the body from various harmful substances [1, 2]. In the wound site, the production and accumulation of reactive oxygen species, prolonged inflammation, and slow cell proliferation are factors that affect wound repair [3, 4]. In recent years, wound management has become a major clinical challenge due to the inherent complexity of traumatic wounds and the current lack of ideal treatment strategies [5, 6]. Different hydrogel-based therapeutic systems have been developed to promote wound healing, and significant results have been achieved at the animal level [7, 8]. However, due to the complexity of the wound, the therapeutic effect of hydrogels on wound healing remains unsatisfactory [9, 10].

Nanoenzymes are prepared, size-controlled, and functionally tunable nanomaterials with enzyme-catalyzed properties [2, 11]. It has been successfully designed to efficiently and stably mimic the activity of natural enzymes [12]. As the research on nanoenzymes progressed, their application potentials in anti-inflammatory, antioxidant damage, and cancer therapy have been extensively explored [13–16]. Several studies reported that MoSe_2_ can serve as a nanoenzyme to scavenge free radicals from damaged tissues and reduce the inflammatory response, thus showing great promise in wound healing research [17, 18]. However, efficiently delivering such nanoenzymes to the target sites is challenging. Platelet-rich plasma (PRP) as a blood-derived product, contains platelets, a small amount of white blood cells and red blood cells. Notably, PRP also contains a variety of cytokines and adhesion molecules that promote migration, adhesion, proliferation and differentiation. In addition to vascular endothelial growth factor (VEGF) and epidermal growth factor (EGF), there is platelet-derived growth factor, basic fibrobast growth factor and transforming growth factor-β [19]. There have been some previous studies on the use of RRP in wound repair, for example, the efficacy of PRP in improving wound healing after maxillofacial surgery was investigated, and the effects were observed by assessing inflammatory factor levels and cellular infiltration [20]. However, PRP has certain drawbacks, such as the burst release, the instability and susceptibility to degradation of growth factors, both of which can limit the long-term application of PRP in damaged tissues [21, 22].

As a hydrophilic substance with a three-dimensional crosslinking structure, hydrogels can rapidly absorb tissue exudate and maintain a moist wound environment for ideal cell attachment and oxygen penetration into damaged tissue to accelerate wound healing [7, 23]. Carboxymethyl chitosan (CMCS) is a water-soluble chitosan derivative with good biocompatibility and biodegradability, which has been found to significantly promote the proliferation of normal skin fibroblasts and effectively accelerate the wound repair process, as well as analgesia, and scar inhibition [24–26]. The hydrogel skeleton made up of CMCS provides a solid foundation for the delivery of nanoenzymes, growth factors, and subsequent treatment; we are therefore eager to design a new type of wound healing material that, in addition to being easy to manufacture and low-cost, should also be able to locally inhibit the excessive production of free radicals, trigger the long-term secretion of growth factors, and promote cell growth and proliferation.

**Scheme 1:**
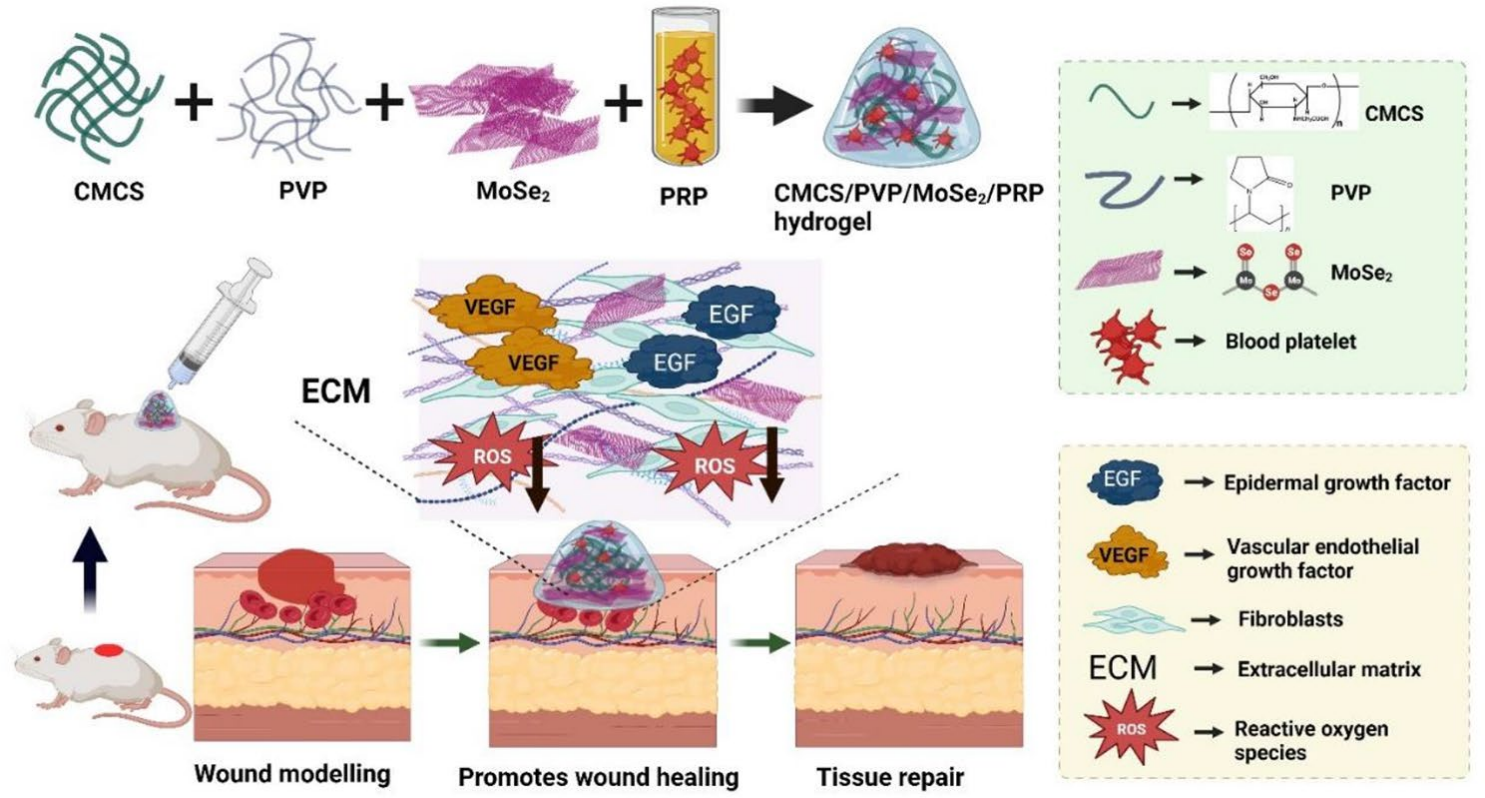
Schematic representation of the multifunctional CMCS/PVP/MoSe_2_/PRP hydrogels for scavenging free radicals from the wound site and releasing multiple factors to promote wound healing (Created by Biorender.com)

In this study, we prepared multifunctional hydrogels (CMCS/PVP/MoSe_2_/PRP hydrogels) using CMCS as the hydrogels backbone and doping with MoSe_2_ nanoenzyme and PRP. Polyvinyl pyrrolidone (PVP), which can form hydrogen bonds with tissue and increase the adhesion properties of hydrogels, was introduced to improve tissue adhesion. Our data proved that the hydrogels exhibited adhesion, appropriate swelling ratio, and biodegradation properties. The MoSe_2_ nanoenzyme in the hydrogels could scavenge ROS from the wound site, conferring excellent antioxidant capacity to the hydrogels. CCK-8, live/dead cell fluorescence staining, and hemolysis assays showed that the CMCS/PVP/MoSe_2_/PRP hydrogels had high cell safety. Cell scratch assays showed that CMCS/PVP/MoSe_2_/PRP hydrogels could promote the proliferation and migration of L929 fibroblasts *in vitro*. Further, we established a whole skin defect model using Balb/c mice to evaluate the effect of CMCS/PVP/MoSe_2_/PRP hydrogels in promoting wound healing (Scheme 1). To the best of our knowledge, the use of MoSe_2_ nanoenzyme-based hydrogel to promote wound healing has not been reported.

## Experimental section

### Materials

CMCS was purchased from Shanghai Macklin Biochemical Technology Co., Ltd. (Shanghai, China). 1-ethyl-3-(3-dimethyl aminopropyl) carbodiimide hydrochloride (EDC), N-hydroxysuccinimide (NHS), and Salicylic acid were purchased from Shanghai Aladdin Reagent Co., Ltd. (Shanghai, China). PVP was purchased from J&K Scientific Biochemical Technology Co., Ltd. (Beijing, China). Hydrogen peroxide was purchased from Sinopharm Chemical Reagent Co., Ltd. (Shanghai, China). Luria-Bertani (LB) was purchased from Yuanye Biotechnology Co., Ltd. (Shanghai, China). Human epidermal growth factor (commercial) was purchased from Guilin Huanowei Gene Pharmaceutical Co. (Guilin, China). 3,3’,5,5’-Tetramethylbenzidine (TMB) was purchased from Shanghai Yuanye Bio-Technology Co., Ltd. (Shanghai, China). Mouse fibroblast cells (L929) were bought from the Institute of Biochemistry and Cell Biology, Chinese Academy of Sciences (Shanghai, China). Dulbecco’s Modified Eagle Medium (DMEM) and phosphate buffer saline (PBS) were procured from Gibco Co., Ltd. (Shanghai, China). The Cell counting kit-8 (CCK-8) was purchased from Wellbio Biotechnology Co., Ltd. (Shanghai). Calcein-AM/P.I. Live/Dead kit was purchased from Solarbio Technology (Beijing) Co., Ltd. Kunming (KM) mice (female, 4–6 weeks, 20–25 g) were ordered from Shanghai Slac Laboratory Animal Center (Shanghai, China). All chemicals were used without further purification. All experimental mice were housed under the management of the Experimental Animal Centre of Changhai Hospital, Naval Medical University. All animal experiments were performed in strict compliance with the protocols approved by the Ministry of Health of the People’s Republic of China and the policies of the Ministry of Health.

### Preparation of hydrogels

To prepare PRP, the collected blood was separated according to the methods reported in the literature [27, 28], MoSe_2_ was prepared according to a previous study [29]. To prepare the hydrogels, 0.1 g of CMCS powder was first dissolved in 2 ml of deionized water in a water bath at 50^◦^C and stirred to form a homogeneous CMCS solution, which was then transferred to a magnetic stirrer for homogenous stirring. Then, the activators of NHS and EDC were dissolved in 1 mL of the MoSe_2_ aqueous solution (100 mg/mL). After the mixture had been wholly dissolved, it was poured into the CMCS solution with continued stirring. Immediately after that, the PVP solution containing 15% PRP was added to the CMCS solution, and by slow stirring and standing, the CMCS/PVP/MoSe_2_/PRP hydrogels were obtained. Based on the above steps, we investigated the synthesis of hydrogels under three different parameters: CMCS/PVP(1): 2 mL of CMCS solution (0.05 g/mL), 1 mL of EDC/NHS solution (0.01 g/mL), and 1 mL of PVP solution (0.2 g/mL); CMCS/PVP(2): 2 mL of CMCS solution (0.05 g/mL), 1 mL of EDC/NHS solution (0.02 g/mL), and 1 mL of PVP solution (0.2 g/mL); CMCS/PVP(3): 2 mL of CMCS solution (0.05 g/mL), 1 mL of EDC/NHS solution (0.02 g/mL), 2 mL of PVP solution (0.2 g/mL). CMCS/PVP/PRP and CMCS/PVP/MoSe_2_ hydrogels were prepared based on CMCS/PVP (3) without adding MoSe_2_ and PRP, respectively. During the experiments, the digital camera recorded all the images of the hydrogels macroscopic gel formation process.

### Characterizations of hydrogels

We used scanning electron microscopy (SEM, Zeiss Sigma 300) to observe the microstructure of hydrogels. The hydrogels were first frozen in a refrigerator before being dried in a lyophilizer and further vacuum dried for 24 h. The solvent in the hydrogels was then wholly evaporated. Next, the dried hydrogels were cut and fixed to the SEM sample stage utilizing conductive adhesive, exposing the cut sections and spraying them with gold. The pore structure of the individual hydrogels was then observed using SEM. Further, the homogeneous distribution of C, O, Se, and Mo in CMCS/PVP/MoSe_2_/PRP hydrogels was recorded by elemental mapping images. The structural information of the CMCS/PVP/MoSe_2_/PRP hydrogels was scanned and recorded in the range of 4000 –500 cm^− 1^ using Fourier transform infrared spectroscopy (FTIR, NICOLET-380, Thermo Fisher Scientific, USA). Finally, the surface chemical state of the doped MoSe_2_ in CMCS/PVP/MoSe_2_/PRP hydrogels was analyzed by X-ray photoelectron spectroscopy (XPS, ESCAlab 250, Thermo Fisher Scientific, USA).

### *In vitro* antioxidant hydrogels swelling studies

The initial weight of the lyophilized CMCS/PVP hydrogels was first weighed (W_1_). The hydrogels were then immersed in PBS buffer and incubated in a 37 °C incubator (*n* = 3). Finally, we removed the hydrogels from the PBS at the experimental time point, carefully removed the water from the surface of the hydrogels using filter paper, and again recorded the final weight of the hydrogels (W_2_). The swelling kinetic curves of hydrogels were obtained from the data, and the swelling ratio of the hydrogels at swelling equilibrium was calculated from Eq. (1).

1 $$\text{S}\text{w}\text{e}\text{l}\text{l}\text{i}\text{n}\text{g} \, \text{r}\text{a}\text{t}\text{i}\text{o}=\frac{W2-W1}{W1}$$

### Degradation of hydrogels

We investigated the *in vitro* biodegradability of CMCS/PVP/MoSe_2_/PRP hydrogels by enzymatic degradation experiments. The initial weight of the lyophilized CMCS/PVP/MoSe_2_/PRP hydrogels was recorded as W_a_. The hydrogels were then immersed in PBS containing 1000 U/mL lysozyme and water containing 1000 U/mL lysozyme (*n* = 3), incubated in a shaker at 100 rpm, 37 °C, and removed at days 1, 3, 7, 14, and 21. The hydrogels were lyophilized and weighed as W_b_. The degradation rate was calculated using the formula.

2 $$\text{W}\text{e}\text{i}\text{g}\text{h}\text{t} \, \text{r}\text{e}\text{m}\text{a}\text{i}\text{n}\text{i}\text{n}\text{g} \, \text{r}\text{a}\text{t}\text{i}\text{o} \, \left({\%}\right) =\frac{{W}_{b}}{{W}_{a}}\times 100\%$$

### Analysis of the mechanical properties of hydrogels

We measured the mechanical properties using a universal material tester (Zwick Roell Z2.5 T.H. with 2.5 kN sensor). The adhesion tensile test of CMCS hydrogels, CMCS/PVP hydrogels, and CMCS/PVP/MoSe_2_/PRP hydrogels (width: 2.5 cm, length: 3 cm, thickness: 0.3 cm) was tested first. The two pigskin (6 cm long and 3 cm wide) ends were stuck with the hydrogels, fixed to the test apparatus, and tested by tensile loading at 2 mm/min. The maximum tensile strength of the hydrogels for adhesion can be determined when the ends of the pigskin are separated. Further, the hydrogels were tested in compression tests by making cylindrical CMCS hydrogels, CMCS/PVP hydrogels, and CMCS/PVP/MoSe_2_/PRP hydrogels of 1.0 cm in height and 1.6 cm in diameter. To determine their compressive properties, the hydrogels were compressed to 80% of their maximum deformation at a predetermined compression rate of 1 mm/min. All these tests were repeated three times in parallel to obtain the final tensile and compressive stress-strain curves and to calculate the average tensile strength.

### Antioxidant properties of hydrogels

#### ·OH scavenging ability

The scavenging ability of CMCS/PVP/MoSe_2_/PRP hydrogels for ·OH was determined using the salicylic acid (SA) method. Specifically, CMCS/PVP/MoSe_2_/PRP hydrogels doped with different concentrations of MoSe_2_ (0, 25, 50, and 100 mg/mL) were mixed with FeSO_4_·7H_2_O (800 µL, 9 mM), ethanolic salicylic acid (800 µL, 9 mM) and H_2_O_2_ (800 µL, 8.8 mM). The solutions were incubated at 37 °C for 30 min in a constant temperature incubator (*n* = 3). The supernatant of these solutions was then collected, and the absorbance curves at 400–800 nm were recorded using a UV-Vis-NIR spectrometer (U-3900 Shimadzu, Japan). The color changes of the solutions at different concentrations were further recorded with a digital camera. The ·OH scavenging ratio was calculated as shown in Eq. (3):

3 $$\text{S}\text{c}\text{a}\text{v}\text{e}\text{n}\text{g}\text{i}\text{n}\text{g} \, \text{r}\text{a}\text{t}\text{i}\text{o} \, \left({\%}\right) =\frac{{\varDelta A}_{0}-{\varDelta A}_{x}}{{\varDelta A}_{0}}\times 100\%$$

In Eq. (3), ΔA_0_ and ΔA_x_ are the absorbance change values of CMCS/PVP/PRP hydrogels in the control group and CMCS/PVP/MoSe_2_/PRP hydrogels, respectively.

#### O_2_^·^^-^ scavenging ability

We used Electron spin resonance (ESR, ELEXSYS II, Bruker, Germany) spectroscopy to examine the ability of CMCS/PVP/MoSe_2_/PRP hydrogels to scavenge O_2_·^−^. Specifically, O_2_·^−^ was generated by mixing xanthine (5 mM) with 10 µL of xanthine oxidase (0.1 U/mL) in 50 mM of PBS. 5,5-dimethyl-1-pyrroline N-oxide (DMPO) as a superoxide anion trapping agent was added to form the adduct DMPO/·OOH^−^. Finally, adducts were mixed with CMCS/PVP/PRP hydrogels and CMCS/PVP/MoSe_2_/PRP hydrogels (MoSe_2_: 25, 50, and 100 mg/mL). The solutions were transferred to sample detection tubes to record ESR signals immediately after incubation in an oven at 37 °C for 8 min (*n* = 3).

#### DPPH scavenging ability

DPPH (1,1-diphenyl-2-picrylhydrazyl, 1.0 mg) was dissolved in 32 mL of anhydrous ethanol. Then, 2 mL of DPPH solution was mixed with 100 mg of CMCS/PVP/PRP and CMCS/PVP/MoSe_2_/PRP hydrogels (MoSe_2_: 25, 50, and 100 mg/mL) and incubated in an oven at 37 °C for 15 min (*n* = 3). Finally, they were placed in a UV-Vis spectrophotometer for spectral scanning in the wavelength range of 400–800 nm, and the color changes of the corresponding solutions were recorded with a digital camera.

#### Peroxidase (POD) scavenging ability

The POD activity was investigated using the TMB method. In this experiment, we used a reaction solution containing H_2_O_2_ (1000 µL, 144 mM) and an equal volume of TMB (3.2 mM) mixed with 100 mg of CMCS/PVP/PRP hydrogels and CMCS/PVP/MoSe_2_/PRP hydrogels (MoSe_2_: 25, 50, and 100 mg/mL). The POD-like activity was investigated by recording the reaction system’s absorbance changes using UV-Vis spectroscopy (*n* = 3).

#### 3-oxo-2-phenyl-4,4,5,5-tetramethylimidazolidine-1-oxide (PTIO) scavenging ability

Specifically, 3 mg of PTIO powder was added to 30 mL of deionized water. The PTIO working solution was homogenously dissolved by sonication. Then, 2 mL of PTIO working solution was mixed with 100 mg of CMCS/PVP/PRP hydrogels and CMCS/PVP/MoSe_2_/PRP hydrogels (MoSe_2_: 25, 50 and 100 mg/mL) and incubated in an oven at 37 °C for 15 min, and finally, the change in absorbance was scanned by UV-Vis spectroscopy (*n* = 3), and the color change of the solution was photographed.

### *In vitro* release of growth factors

We placed CMCS/PVP/MoSe_2_/PRP hydrogels in 50 mL of centrifuge tubes with 5 mL of PBS buffer solution (*n* = 3). At predetermined time points (0.5 h, 2 h, 6 h, 8 h, 12 h, 24 h, 48 h, and 72 h), 250 µL of sample solution was collected from the centrifuge tubes and replaced with the same volume of PBS. The collected sample solution was stored in the refrigerator at -80 °C. Finally, the concentrations of EGF and VEGF in the sample solution were measured using the enzyme-linked immunosorbent assay (ELISA) kit according to the manufacturer’s instructions.

### Examination of *in vitro* cell migration properties

We studied the effect of CMCS/PVP/MoSe_2_/PRP hydrogels on the migration of mouse fibroblasts (L929 cells) using a cell scratch assay. The CMCS/PVP/MoSe_2_/PRP hydrogels were first sterilized and co-incubated in DMEM medium at 37 °C for 24 h (hydrogels: DMEM medium = 1:10) to obtain the hydrogels extracts and gradient diluted to the desired concentrations (0.25, 0.5, and 1 mg/mL). L929 cells were inoculated into 6-well plates at a cell density of 4 × 10^4^ per well to form a fused monolayer. When the cells were uniformly distributed over the healthy plates at about 80% coverage, the monolayer was scratched with a 20-µL pipette tip to simulate a wound and then incubated with low serum medium for 24 h. After preparation, L929 cells were gently washed twice with PBS to remove cell debris while avoiding blowing up the adherent cells. Finally, 2 mL of hydrogel extracts was added to the above healthy plates, and incubation was continued in an incubator containing 5% CO_2_ at 37 °C. Cell migration was observed at 0 h, 12 h, and 24 h, respectively, and photographed with an inverted microscope (DMI4000B. Leica, Germany). Finally, the scratch area at each time point was quantified according to the pictures using ImageJ software. The cell migration ratio was calculated as follows (A_0_: the initial scratch area at 0 h, A_t_: the remaining scratch area after the specified incubation time):

4 $${\text{Cell}} \, {\text{migration}} \, {\text{ratio}}\left({\%}\right) =\frac{{A}_{0}-{A}_{t}}{{A}_{0}}\times 100\%$$

### *In vitro* compatibility of CMCS/PVP/MoSe_2_/PRP hydrogels

We first examined the hemocompatibility of the prepared CMCS/PVP/MoSe_2_/PRP hydrogels using whole blood from Kunming (KM) mice provided by the Animal Centre of Changhai Hospital, Naval Medical University. The mice red blood cells (mRBCs) were diluted 100-fold in PBS buffer, and 4 mL of the above PBS-diluted mRBCs suspension was mixed with 5, 25, 50, and 100 mg of CMCS/PVP/MoSe_2_/PRP hydrogels in a centrifuge tube. Similarly, mRBCs suspensions treated with PBS and H_2_O were defined as positive control and positive control, respectively. The above-mixed solutions were incubated at 37 °C for 2 h. Subsequently, the supernatant was collected by centrifugation (5000 rpm, 5 min), and the absorbance values of the solutions were measured at 541 nm. The corresponding hemolysis ratio was calculated using the following formula, and digital photographs of the corresponding solutions were recorded.

5 $$\text{H}\text{e}\text{m}\text{o}\text{l}\text{y}\text{t}\text{i}\text{c} \, \text{r}\text{a}\text{t}\text{i}\text{o} \left({\%}\right)=\frac{{A}_{s}-{A}_{n}}{{A}_{p}-{A}_{n}}\times 100{\%}$$

Where A_s_ represents the absorbance of the hydrogels-treated mRBCs suspension at 541 nm, A_n_ represents the absorbance of the mRBCs suspension treated with PBS, and A_p_ represents the H_2_O-treated mRBCs suspension.

Further, we determined the cytocompatibility of the hydrogels using the leachate of CMCS/PVP/MoSe_2_/PRP hydrogels. L929 cells were inoculated in 96-well plates (4 × 10^3^ cells/well), and 100 µL of different concentrations of hydrogel extracts was added and co-incubated in a 5% CO_2_ incubator. Cell survival was observed on days 1, 3, and 5, respectively, and images were collected using live/dead cell staining and the cell viability of L929 cells was detected using the CCK-8 kit.

### *In vitro* safety and degradation properties of CMCS/PVP/MoSe_2_/PRP hydrogels

The animal study protocol was approved by the Institutional Review Board of The First Affiliated Hospital of Naval Medical University of the People’s Liberation Army (SYXK(Shanghai) 2020-0033). CMCS/PVP/MoSe_2_/PRP hydrogels (approximately 0.6 cm in diameter and 0.2 cm in thickness, *n* = 3) were prepared in advance using a mold and aseptically treated. The mice were anesthetized and skin was prepared, and the incision sites were disinfected with iodophor. The hydrogels were implanted into the mice’s dorsal skin and then sutured with simple interrupted sutures before resting for observation. The body weight of the mice was monitored before and after the experiment. At the end of the procedure, the mice were executed at 7, 14, and 28 days after feeding, and the subcutaneous hydrogels were extracted, weighed, and photographed to determine the *in vivo* biodegradation. During this procedure, mouse blood was extracted for serum biochemical analysis. Meanwhile, skin tissues of mice exposed to the hydrogels and their major organs (heart, liver, spleen, lungs, and kidneys) were dissected, fixed in paraformaldehyde, and analyzed by hematoxylin-eosin (H&E) staining for tissue analysis. H&E staining images were recorded by a German inverted phase contrast microscope (Leica DM IL LED).

### Assessment of the wound healing capacity

To evaluate the ability of CMCS/PVP/MoSe_2_/PRP hydrogels to scavenge free radicals and promote wound healing, a whole skin wound model was established using Balb/c mice for *in vivo* studies. The experimental procedure was as follows: after disinfecting the experimental table, the mice were anesthetized, and part of their dorsal hair was removed and disinfected with iodophor. The dorsal skin of the mice was gently pulled upwards, and a full-length wound with a diameter of 0.6 cm was made on the dorsal surface of each Balb/c mouse using a disinfected perforator. CMCS hydrogels, CMCS/PVP hydrogels, commercially available gels (human epidermal growth factor), CMCS/PVP/MoSe_2_ hydrogels and CMCS/PVP/MoSe_2_/PRP hydrogels (*n* = 3) were applied to the wounds of the corresponding mice and fixed with sterile gauze. Wound healing in mice was recorded using a digital camera on days 0, 2, 5, 8, 12, and 16, respectively, and the wound area was analyzed using ImageJ software to assess the wound healing-promoting effect of the hydrogels. The change in wound area over time was expressed as the original wound area. The wound healing ratio was calculated as follows.

6 $$\text{W}\text{o}\text{u}\text{n}\text{d} \, \text{h}\text{e}\text{a}\text{l}\text{i}\text{n}\text{g}\, \text{r}\text{a}\text{t}\text{i}\text{o} \left({\%}\right)=\frac{{S}_{s}}{{S}_{p}}\times 100{\%}$$

In the formula, S_s_ and S_p_ are the open and original wound areas on the specified date, respectively. On day 16, different groups of mice were executed, and the skin tissue around the wound was collected and fixed in paraformaldehyde and analyzed histologically to assess wound healing [H&E staining, Masson staining, Sirius red staining, Platelet-endothelial cell adhesion molecule (PECAM-1/CD31) staining and α-Smooth muscle actin (α-SMA) staining]. Immunofluorescence staining images were obtained and viewed by fluorescence microscopy, followed by quantitative analysis by Image J software. The serum (day 16) and skin tissues (day 8 and day16) of the experimental mice were also collected to determine TNF-α, EGF, and VEGF levels using ELISA kits and the double antibody sandwich enzyme-linked immunosorbent assay technique. In brief, standards, samples to be tested, and biotinylated detection antibodies are added to the wells of the enzyme plate and incubated at room temperature for 2 h. After removing unbound agents, horseradish peroxidase-labeled streptavidin (Streptavidin-HRP) was added. After washing, the chromogenic substrate TMB was added. The reaction was terminated when the color changed from blue to yellow by adding a termination solution. The absorbance was measured at 450 nm within 30 min to calculate the TNF-α, EGF, and VEGF levels.

### Statistical analysis

All results were expressed as mean ± SD. Statistical significance was determined using a one-way analysis of variance (ANOVA), and statistically significant differences were marked with *p < 0.05, **p < 0.01, and ***p < 0.001.

## Results and discussion

### Synthesis and selection of parameters for antioxidant hydrogels

**Fig. 1:**
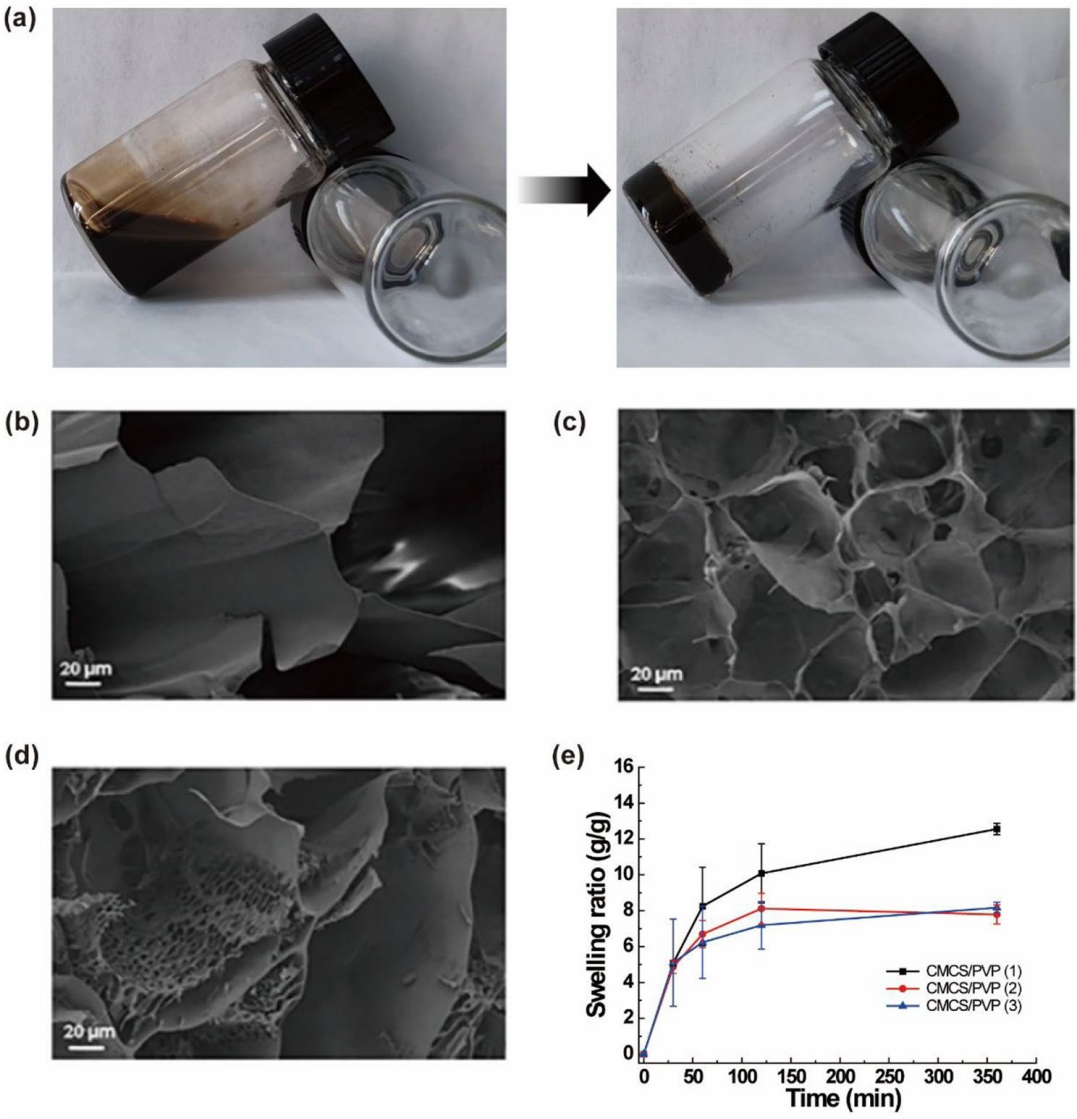
**(a)** Digital images of the hydrogels formation process of CMCS/PVP/MoSe_2_/PRP hydrogels; **(b)**—**(d)** SEM images of CMCS/PVP hydrogels (**(b)**: CMCS/PVP (1); **(c)** CMCS/PVP (2); **(d)** CMCS/PVP (3); **(e)**) swelling ratio of CMCS/PVP hydrogels in PBS

Multifunctional composite hydrogels are rapidly developing in tissue engineering [30]. The hydrogels have a 3D structure that can mimic the natural microenvironment of tissues and control the behavior of cells [7]. In this study, we used the biocompatible and biodegradable CMCS as the hydrogel backbone and crosslinked it under the activation of EDC/NHS to form hydrogels. To improve the adhesion properties, PVP was introduced. Finally, MoSe_2_ nanoenzyme with antioxidant ability and PRP that is capable of releasing growth factors were added to the precursor solution for hydrogel formation. The macroscopic changes of hydrogels synthesis process are shown in Fig. 1. The precursors with a mobile state underwent polymerization to form solid CMCS/PVP/MoSe_2_/PRP hydrogels under the activation of EDC and NHS (Fig. 1a). The typical three-dimensional structure of interconnected pores could be observed in all the CMCS/PVP hydrogels. Besides, the hydrogels showed successive decreases in pore size as the EDC/NHS equivalents increased (Fig. 1b-d). In addition, we performed the swelling ratio of hydrogels (Fig. 1e). The results showed that the swelling ratio of the hydrogels with each parameter in PBS solution was CMCS/PVP (1): 12.55 ± 0.31 g/g, CMCS/PVP (3): 7.78 ± 0.53 g/g and CMCS/PVP (2): 8.17 ± 0.3 g/g. Considering the pore size and swelling effect, for further studies, CMCS/PVP/MoSe_2_/PRP hydrogel with the MoSe_2_ doping level of 25 mg/mL was selected.

### Characterization evaluation of antioxidant hydrogels

**Fig. 2:**
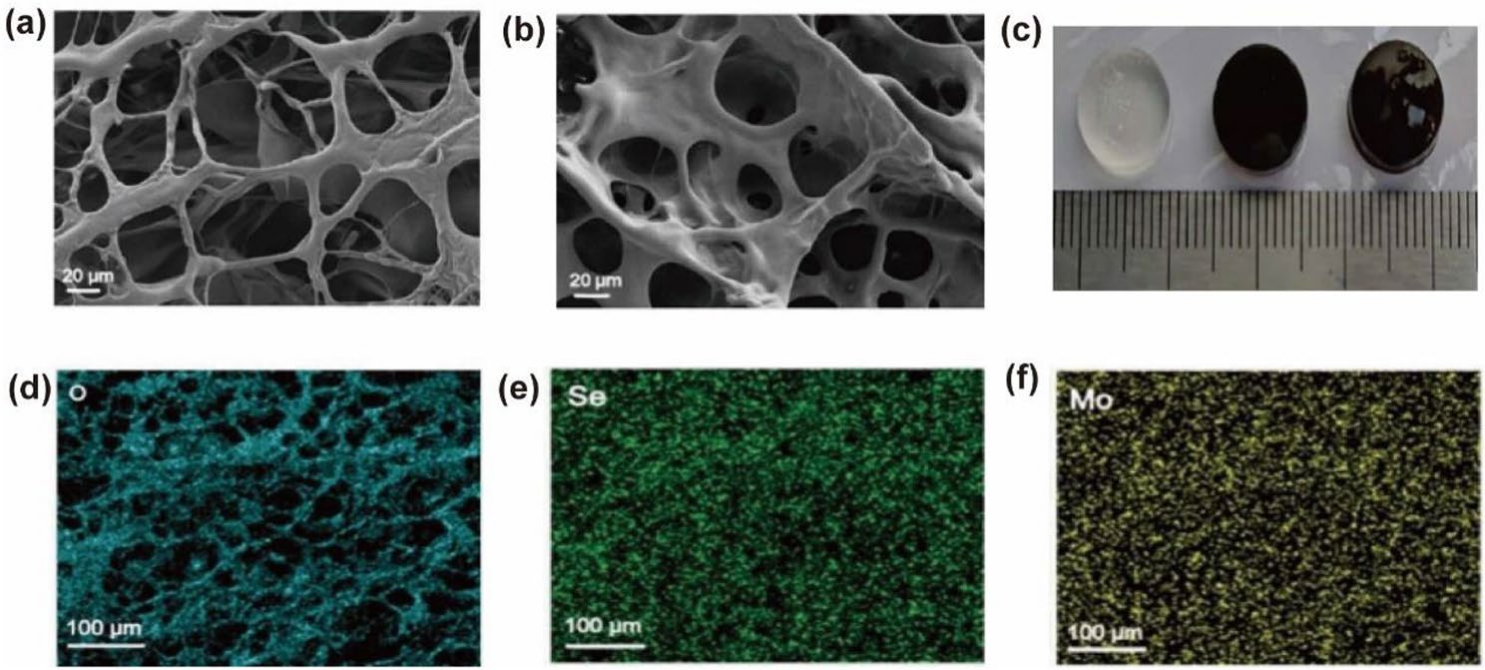
**(a)** SEM image of CMCS/PVP/MoSe_2_ hydrogels; **(b)** SEM image of CMCS/PVP/MoSe_2_/PRP hydrogels; **(c)** digital image of the prepared hydrogels (from left to right: CMCS/PVP hydrogels, CMCS/PVP/MoSe_2_ hydrogels, and CMCS/PVP/MoSe_2_/PRP hydrogels); **(d)**—**(f)** distribution of O, Se and Mo elements in CMCS/PVP/MoSe_2_/PRP hydrogels

**Fig. 3:**
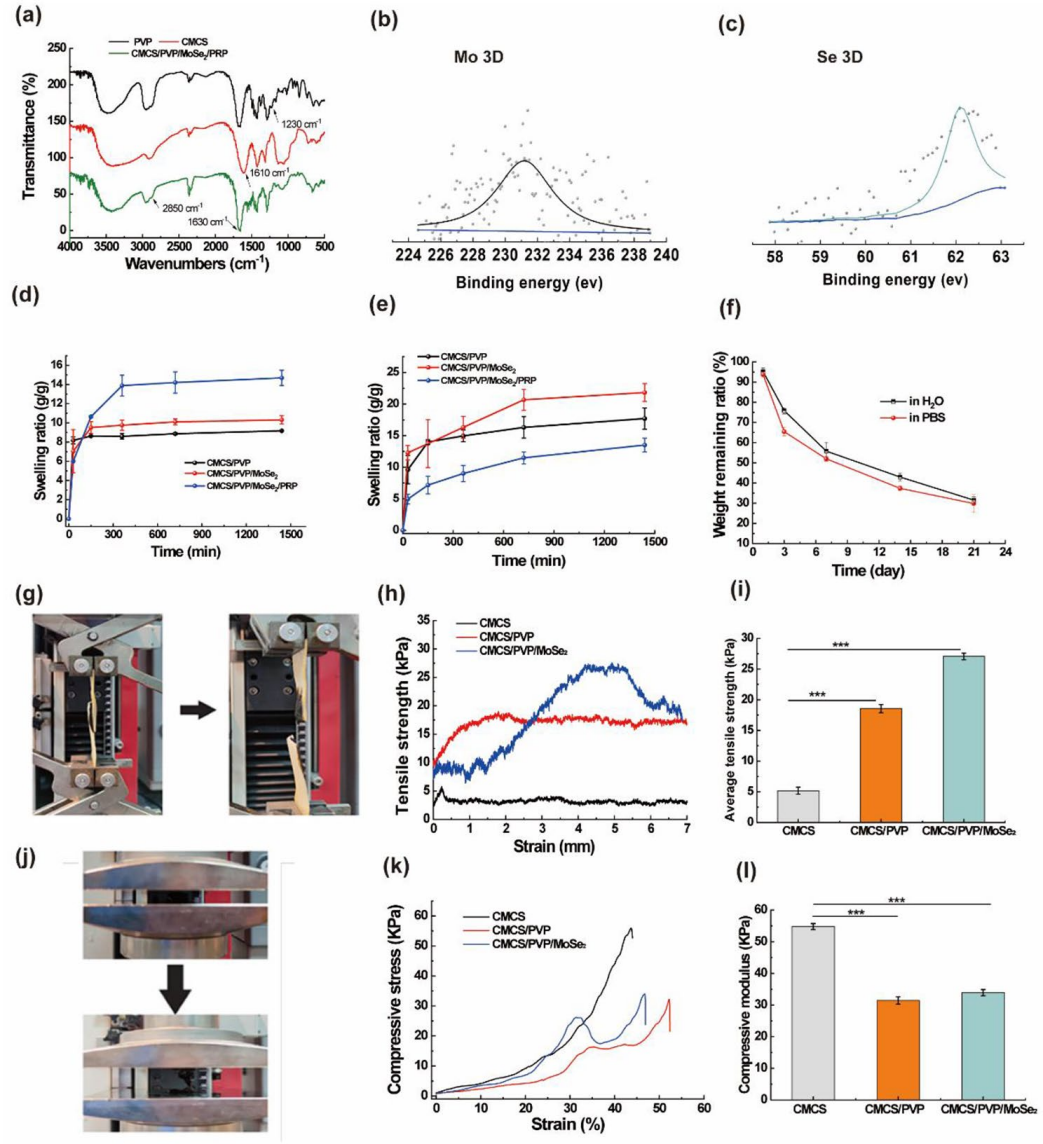
**(a)** FTIR spectra of PVP, CMCS, and CMCS/PVP/MoSe_2_/PRP hydrogels; **(b)** XPS spectrum of Mo 3d; **(c)** XPS spectrum of Se 3d; **(d)** and **(e)** swelling curves of hydrogels in **(d)** PBS and **(e)** H_2_O; **(f)***in vitro* degradation rate curves of CMCS/PVP/MoSe_2_/PRP hydrogels in PBS solution and H_2_O; **(g)** strain-stress test picture (left: before stretching, right: after stretching); **(h)** strain-stress curves for shear tests and **(i)** final average tensile strength; **(j)** macroscopic images of CMCS/PVP/MoSe_2_ hydrogels in compression (top: before compression, bottom: after compression) **(j)** strain-stress curves for compression tests and **(k)** final mean compressive strength of hydrogels

Using SEM, we then observed the morphological characteristics of the CMCS/PVP/MoSe_2_ hydrogels and CMCS/PVP/MoSe_2_/PRP hydrogels. The SEM images of all hydrogels showed interconnected and porous microstructures (Fig. 2a-b). Digital images of the prepared CMCS/PVP hydrogels and CMCS/PVP/MoSe_2_ hydrogels are shown in Fig. 2c. The elemental distribution in Fig. 2d-f shows that O, Se, and Mo elements are uniformly distributed in the CMCS/PVP/MoSe_2_/PRP hydrogels, further indicating that MoSe_2_ is uniformly doped in the CMCS/PVP/MoSe_2_/PRP hydrogels. The formation mechanism of the hydrogels was investigated using FTIR. In the FTIR spectra of the CMCS/PVP/MoSe_2_/PRP hydrogels (Fig. 3a), the characteristic peaks at 1230 cm^− 1^ can be assigned to PVP, at 2850 cm^− 1^ and 1610 cm^− 1^ can be considered as the - N-H bond and the characteristic peak at 1630 cm^− 1^ can be considered as an amide bond (-CO-NH-), confirming that the hydrogels formation was based on the reaction between -COOH and -NH_2_ in CMCS. XPS was used to determine the chemical structure of MoSe_2_, and the XPS spectra of Mo 3d and Se 3d were shown in Fig. 3b-c. The characteristic peaks at 231.2 eV and 62.1 eV indicated that the doping process did not change the elemental state of MoSe_2_.

### Evaluation of the swelling properties and degradability of hydrogels

The swelling property is one of the essential physical parameters of the hydrogels, which affects the release rate of the loaded compound and the uptake of tissue exudate [31]. As can be seen from Fig. 3d-e, the equilibrium swelling ratio of hydrogels gradually increased with the addition of MoSe_2_ and PRP in PBS and H_2_O (Fig. 3d-e). Specifically, the equilibrium swelling ratio of CMCS/PVP hydrogels, CMCS/PVP/MoSe_2_ hydrogels, and CMCS/PVP/MoSe_2_/PRP hydrogels in PBS were gradually increased with the incorporation of MoSe_2_ and PRP, and the equilibrium swelling ratios were calculated to be 920%, 1031%, and 1469%, respectively. Correspondingly, the equilibrium swelling ratio of CMCS/PVP/MoSe_2_/PRP hydrogels reached 1350% in H_2_O. The above results indicate that after the addition of PRP and MoSe_2_, the pore size distribution is reduced, the hydrogels become dense, and the absorbed water does not dissipate as quickly as in CMCS/PVP, which improves the swelling ratio. This excellent swelling ability will facilitate the absorption of wound exudate, which is essential for cell growth, proliferation, and migration during the wound-healing process. Next, we investigated the degradation of CMCS/PVP/MoSe_2_/PRP hydrogels in H_2_O and PBS. The remaining weight ratio of CMCS/PVP/MoSe_2_/PRP hydrogels degraded on day 21 was 31.53% in H_2_O and 29.85% in PBS (Fig. 3f), suggesting the hydrogels has an appropriate degradation ratio, which could afford for the gradual release of the loaded substances.

### Analysis of the mechanical properties of hydrogels

An ideal wound dressing material requires appropriate mechanical strength and flexibility. Therefore, we tested the hydrogels’ mechanical properties through lap shear and compression tests. The macroscopic images of the tensile experimental process of the hydrogels are shown in Fig. 3g. The tensile stress-strain curves (Fig. 3h) showed that the CMCS/PVP/MoSe_2_ hydrogels have a significantly higher adhesive tensile strength compared to CMCS hydrogels and CMCS/PVP hydrogels, which indicates that PVP improved the adhesive properties of the hydrogels and that the addition of MoSe_2_ does not affect the adhesion. As shown in Fig. 3h-i, the tensile data likewise indicate that doping with PVP leads to an increase in shear property, which is reflected in the final average tensile strength data of the hydrogels (Fig. 3i). The corresponding compression images are shown in Fig. 3j. The compressive strengths of CMCS/PVP/MoSe_2_ hydrogels and CMCS/PVP hydrogels are lower than those of CMCS hydrogels, with average compressive strengths of 33.9 kPa, 31.4 kPa, and 54.8 kPa, respectively (Fig. 3k-l). This shows that in contrast to the brittleness of the CMCS hydrogels, the addition of PVP and MoSe_2_ increased the flexibility of the hydrogels, which is mainly attributed to the increased hydrogen bonding.

### Evaluation of the antioxidant properties of hydrogels

**Fig. 4:**
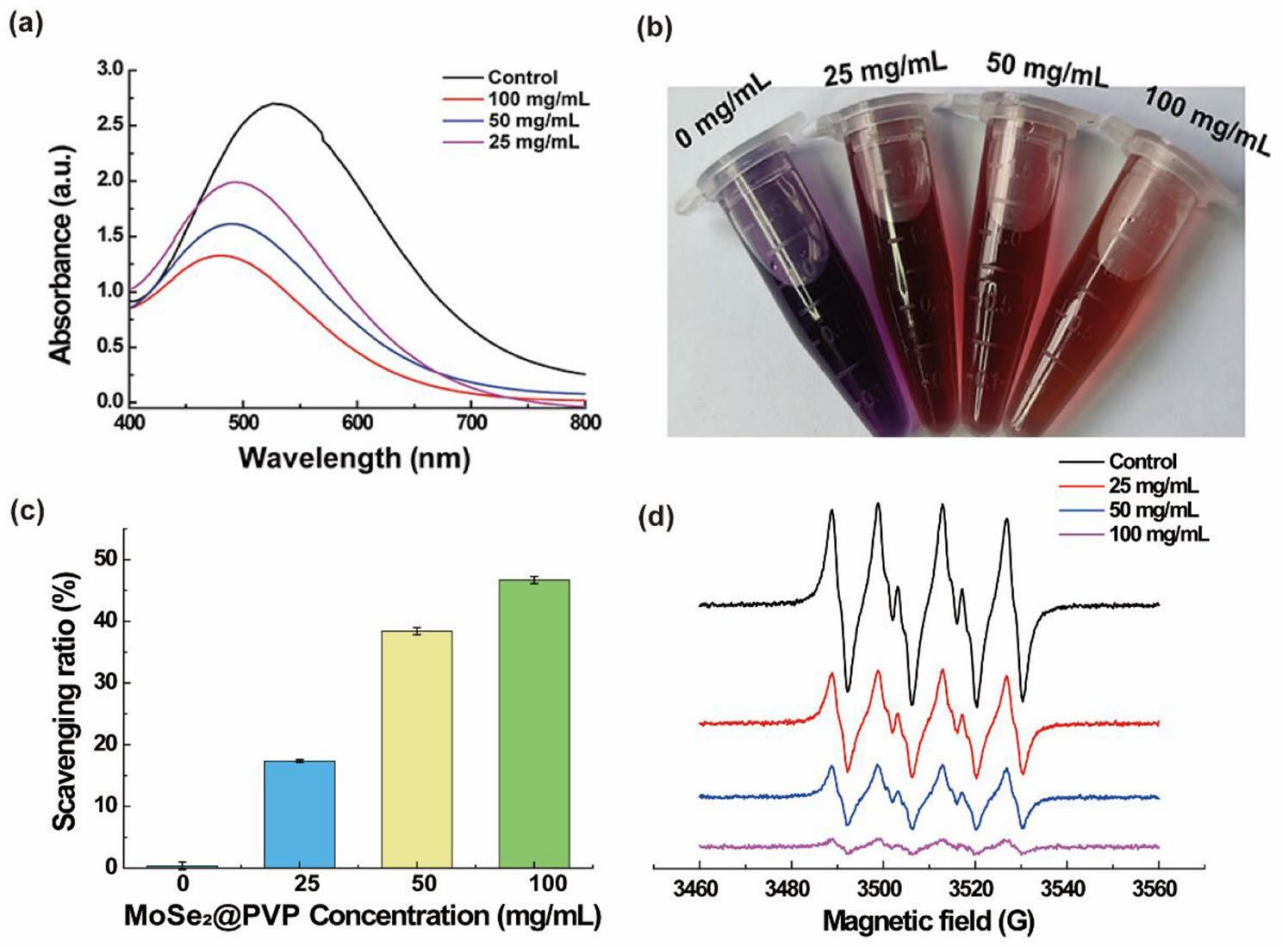
**(a)** UV-Vis absorption spectra of salicylic acid without (control) and with CMCS/PVP/MoSe_2_/PRP hydrogels; **(b)** digital photographs of the co-incubated solutions corresponding to **(a)**; **(c)** scavenging ratio of ·OH radicals by CMCS/PVP/MoSe_2_/PRP hydrogels; **(d)** ESR spectra of DMPO/O_2_·^−^ after incubated with CMCS/PVP/MoSe_2_/PRP hydrogels

As wounds generate many free radicals and undergo oxidative stress, biomaterials with good antioxidant capacity may thus accelerate wound healing. The scavenging ability of CMCS/PVP/MoSe_2_/PRP hydrogels for ·OH was first evaluated using the SA method. In this method, the ·OH radical reacts with SA to form a purple 2,3-dihydroxybenzoic acid with a specific absorption at 510 nm. Therefore, the ·OH scavenging ability of CMCS/PVP/MoSe_2_/PRP hydrogels can be assessed by measuring the absorbance of the reaction solution at 510 nm. As shown in Fig. 4a-b, SA was readily oxidized by ·OH and produced a purple solution without the participation of CMCS/PVP/MoSe_2_/PRP hydrogels. However, adding CMCS/PVP/MoSe_2_/PRP hydrogels resulted in a significant absorption decrease at 510 nm, and the color of the corresponding reaction solution gradually decreased. Moreover, the absorption decrease is concentration-dependent (Fig. 4a-b). When the doping concentration of MoSe_2_ in the CMCS/PVP/MoSe_2_/PRP hydrogels was 100 mg/mL, the ·OH radical scavenging ratio reached 47.1% (Fig. 4c), revealing that the CMCS/PVP/MoSe_2_/PRP hydrogels had specific ·OH scavenging activity.

**Fig. 5:**
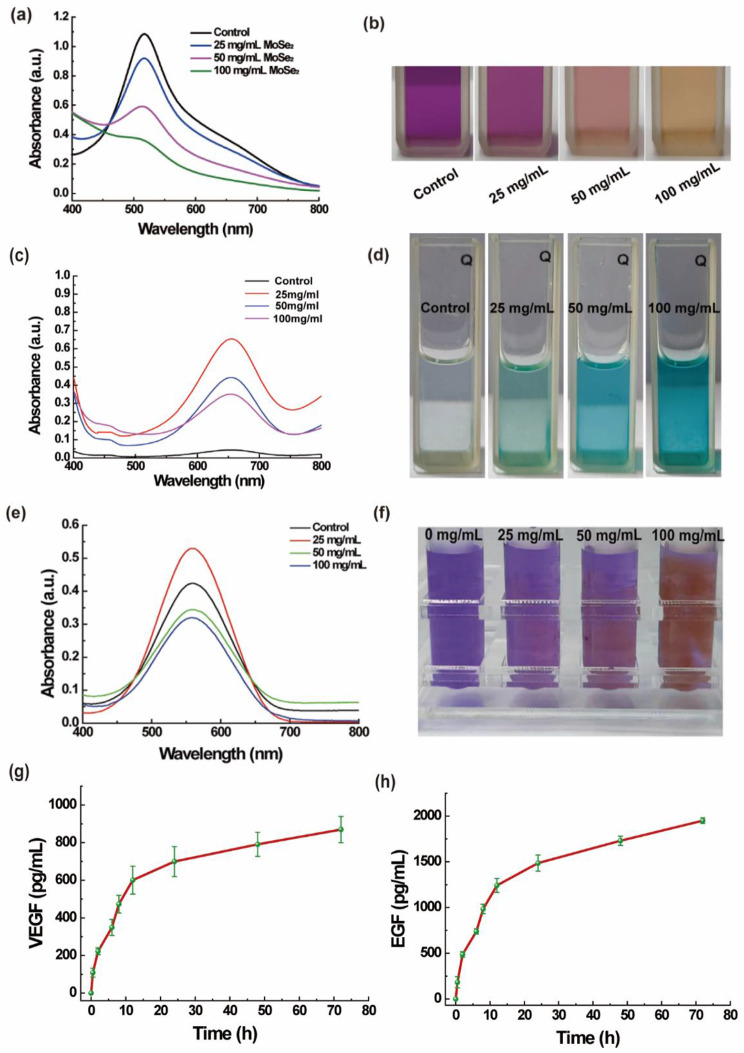
**(a)** UV-Vis spectra of the DPPH solution after co-incubation of CMCS/PVP/MoSe_2_/PRP hydrogels with different MoSe_2_ concentrations; **(b)** digital images of the solution corresponding to **(a)**; **(c)** UV-Vis spectra of the solution after co-incubation of TMB with CMCS/PVP/MoSe_2_/PRP hydrogels in the presence of H_2_O_2_; **(d)** digital images of the solution corresponding to **(c)**; **(e)** UV-Vis spectra of CMCS/PVP/MoSe_2_/PRP hydrogels solution after co-incubation with PTIO; **(f)** digital images of the solutions corresponding to **(e)**; **(g)** and **(h)** release profile of **(g)** VEGF and **(h)** EGF from CMCS/PVP/MoSe_2_/PRP hydrogels

Apart from the ·OH, O_2_·^−^ is also capable of causing oxidative stress-related diseases. Therefore, we examined the performance of CMCS/PVP/MoSe_2_/PRP hydrogels to scavenge O_2_·^−^ using the ESR technique. As shown in Fig. 4d, the control group generated a classical superoxide radical six-fold peak. In sharp contrast, with the addition of CMCS/PVP/MoSe_2_/PRP hydrogels, the intensity of the characteristic peak decreased rapidly with a concentration-dependent elimination trend. The DPPH method was further used to determine antioxidant capacity. DPPH radical has a strong absorption at 520 nm. Substances with antioxidant capacity can react with the DPPH radical and decrease the solution absorbance at 520 nm. In our experiments, with the addition of MoSe_2_ in the hydrogels, the intensity of the absorption peak corresponding to DPPH at 520 nm gradually decreased. The peak intensity is negatively correlated with the doping concentration of MoSe_2_ in the hydrogels (Fig. 5a). The color of the corresponding solution also faded gradually, indicating that the CMCS/PVP/MoSe_2_/PRP hydrogels have a good DPPH scavenging ability (Fig. 5b).

POD is widely distributed in cells, which catalyzes substrate oxidation with H_2_O_2_ eliminates the H_2_O_2,_ and reduces phenolic amine toxicity. We used TMB as a substrate to test the ability of CMCS/PVP/MoSe_2_/PRP hydrogels to mimic POD activity. As shown in Fig. 5c, the peak of oxidized TMB gradually increased at 650 nm with increasing concentrations of MoSe_2_ in the hydrogels. In addition, the color change of the corresponding solution confirmed the POD mimetic activity of CMCS/PVP/MoSe_2_/PRP hydrogels (Fig. 5d).

Furthermore, NO, an essential biomolecule, plays a vital role in the development of many diseases. The reaction of PTIO with NO results in the formation of imino nitrogen oxides, a reaction that can be used to examine the inhibitory activity of NO synthase. Specifically, the free radical solution of PTIO is violet in color with a characteristic absorption peak at 560 nm, which lightens in color and decreases in the absorbance value due to the scavenging of the free radicals. Therefore, it was used to study the antioxidant ability of CMCS/PVP/MoSe_2_/PRP hydrogels. As shown in Fig. 5e-f, the highest absorbance value at 560 nm was observed in the control group. When CMCS/PVP/MoSe_2_/PRP hydrogels were added, the absorbance value gradually decreased, and the purple color of the solutions faded and showed a MoSe_2_ concentration dependence. These results suggested that CMCS/PVP/MoSe_2_/PRP hydrogels can scavenge a variety of harmful free radicals and have the potential to be used as wound dressings to scavenge free radicals and promote wound healing.

### Evaluation of the release of growth factors from hydrogels

PRP can release various growth factors, which play a crucial role in regulating the proliferation of mesenchymal cells and the production of extracellular matrix [22, 32]. Moreover, it has been shown that PRP is able to inhibit the release of cytokines and inflammation, promote the formation of new capillaries, and accelerate the wound healing and tissue regeneration [21, 33]. However, the burst release of growth factors may lead to uncontrolled tissue regeneration. Therefore, herein, PRP was incorporated into hydrogels to achieve sustained release of growth factors. In our experiments, we used an ELISA kit to detect whether PRP-loaded CMCS/PVP/MoSe_2_/PRP hydrogels could sustain the release of growth factors, by selecting EGF and VEGF as representatives. The results showed that the release behaviors of VEGF and EGF were linear during the first 24 h, after which the release rate slowed down. The CMCS/PVP/MoSe_2_/PRP hydrogels exhibited prolonged release of growth factors, indicating that the hydrogels can provide a stable supply of growth factors during wound healing and have great potential as a PRP bioactive wound dressing (Fig. 5g-h).

### Evaluation of *in vitro* cell migration performance

**Fig. 6:**
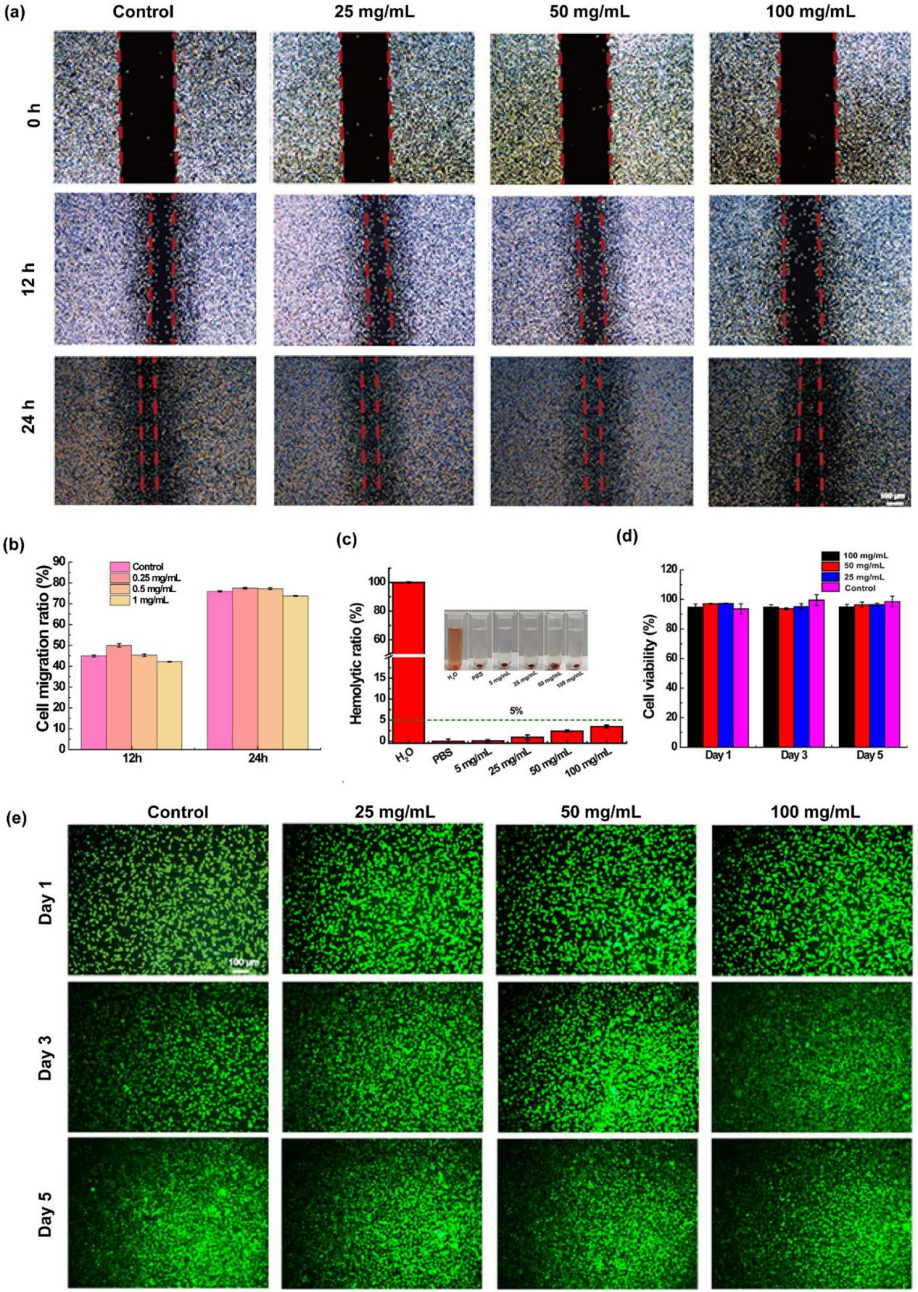
**(a)** Images of L929 cell migration after co-cultured with CMCS/PVP/MoSe_2_/PRP hydrogels; **(b)** cell migration ratio at different time points; **(c)** hemolysis ratio of erythrocytes after treatment with CMCS/PVP/MoSe_2_/PRP hydrogels and digital images of the corresponding solutions; **(d)** CCK-8 results of L929 cells treated with CMCS/PVP/MoSe_2_/PRP hydrogels extracts; **(e)** images of live/dead cell staining of L929 cells after treatment with CMCS/PVP/MoSe_2_/PRP hydrogels extracts

Studies have shown that PRP enhanced cell proliferation and migration, and we used a scratch assay to assess the effect of CMCS/PVP/MoSe_2_/PRP hydrogels on fibroblast L929 migration. The results (Fig. 6a) showed digital images of L929 cell migration at different incubation times after treating CMCS/PVP/MoSe_2_/PRP hydrogels. At 12 h, CMCS/PVP/MoSe_2_/PRP hydrogels (50 mg/mL) had the most potent effect on the migration of L929 cells compared to the other experimental groups (Fig. 6a-b). The CMCS/PVP/MoSe_2_/PRP hydrogels (25 and 50 mg/mL) showed a more effective migration of cells at 12 h (50 ± 0.83% and 45.27 ± 0.6%, respectively) than the control (44.9 ± 0.5%). Scratch wound healing of the hydrogels at 24 h was not significantly different from the untreated cell control, with the scratch area primarily covered by cells. All results indicated that the VEGF and EGF growth factors contained in the CMCS/PVP/MoSe_2_/PRP hydrogels can stimulate cell migration and favor the growth and proliferation of L929 cells, thus promoting wound healing and regeneration.

### *In vitro* compatibility evaluation of hydrogels

Good biocompatibility of the prepared hydrogels is essential for its tissue engineering applications. In the hemo-compatibility assay, it was found that both the negative control (PBS) and different concentrations of CMCS/PVP/MoSe_2_/PRP hydrogels extracts did not affect the erythrocytes, with a hemolysis ratio below 5%. However, the red color after treatment with deionized water (positive control) indicated that significant hemolysis had occurred (Fig. 6c). Furthermore, the CCK-8 assay showed that the cell viability of L929 cells was maintained above 90% after co-cultured with different concentrations of CMCS/PVP/MoSe_2_/PRP extracts (25, 50, and 100 mg/mL) for 1, 3 and 5 days (Fig. 6d). The morphology of L929 cells was further qualitatively evaluated by live/dead cell staining. During the co-incubation of the hydrogel extracts with the cells, the live cells that appeared green in the field of view increased over time (Fig. 6e). In conclusion, the experimental results indicated that the prepared CMCS/PVP/MoSe_2_/PRP hydrogels have good biocompatibility.

### *In vitro* degradation and safety evaluation of hydrogels

**Fig. 7:**
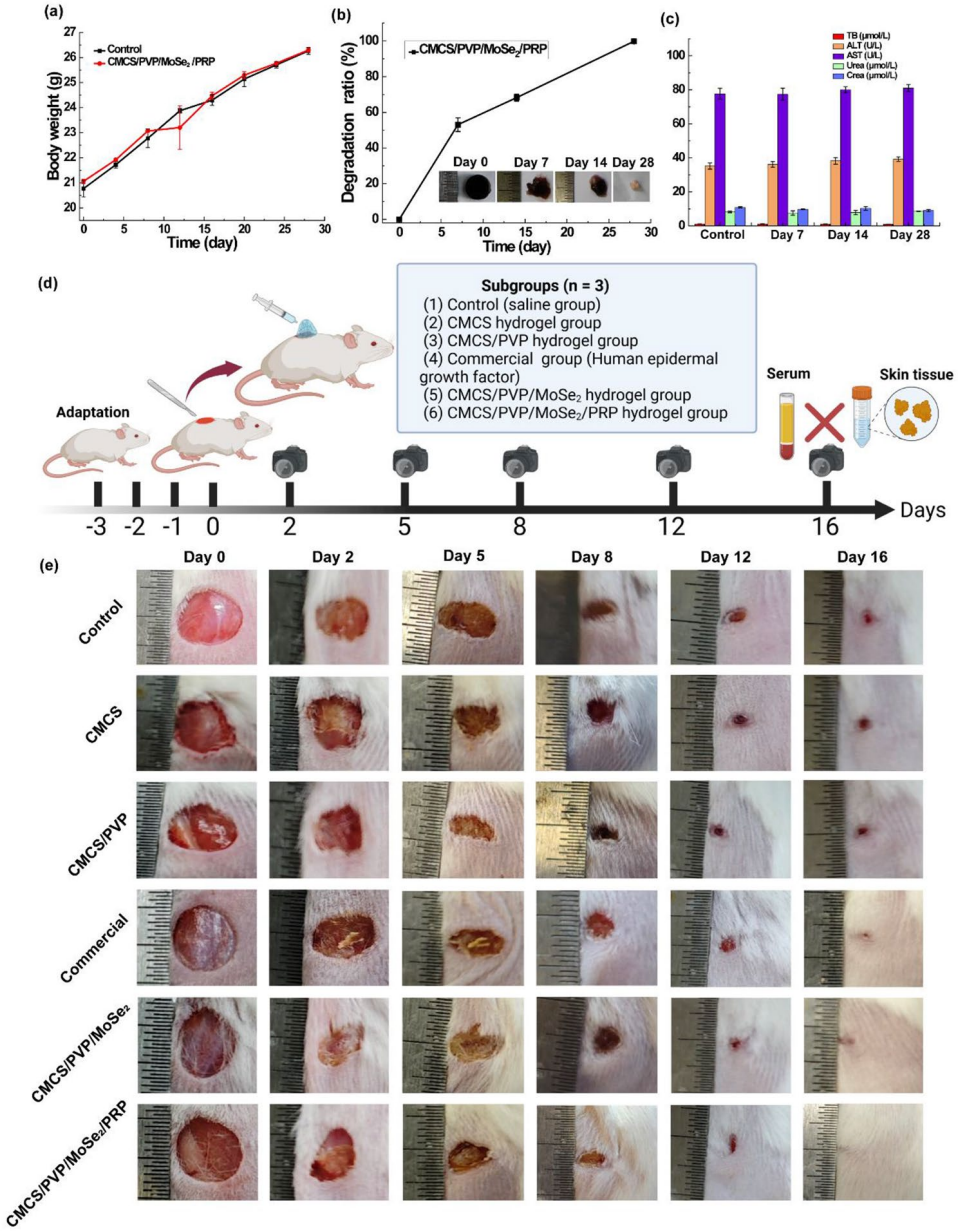
**(a)** Body weight change curves of KM mice after different treatments; **(b)***in vivo* degradation curves of CMCS/PVP/MoSe_2_/PRP hydrogels and corresponding digital images; **(c)** blood biochemical results of KM mice after hydrogels treatment at different time points; **(d)** flow chart of the trauma wound healing experiments (created by Biorender.com); **(e)** representative photographs of wound healing in Balb/c mice treated with different hydrogels on days 0, 2, 5, 8, 12, and 16

After proving the excellent *in vitro* biocompatibility of CMCS/PVP/MoSe_2_/PRP hydrogels, we investigated their safety and degradation properties at the *in vivo* level. The body weight of KM mice was first monitored and recorded every four days. As shown in Fig. 7a, there was no significant difference in body weight change between the CMCS/PVP/MoSe_2_/PRP hydrogels-embedded KM mice and healthy mice, which indicated that the hydrogels did not affect the growth of KM mice. The *in vivo* degradation of the embedded CMCS/PVP/MoSe_2_/PRP hydrogels was measured in KM mice after 7, 14, and 28 days of feeding. The weight of the hydrogels decreased to 53.08 ± 3.85% and 68.24 ± 1.94% on days 7 and 14, respectively. On day 28, the CMCS/PVP/MoSe_2_/PRP hydrogels were degraded, indicating that the hydrogels have good biodegradability (Fig. 7b). In addition, the serum biochemical parameters showed no significant differences between the hydrogels-embedded KM mice and healthy mice (Fig. 7c). The H&E staining results showed no significant pathological differences in the major organs and skin of KM mice (Fig. S2a). The results confirmed that the CMCS/PVP/MoSe_2_/PRP hydrogels have good degradation properties and biosafety, thus providing the desired feasibility for promoting wound healing.

### *In vitro* wound healing evaluation of hydrogels

**Fig. 8:**
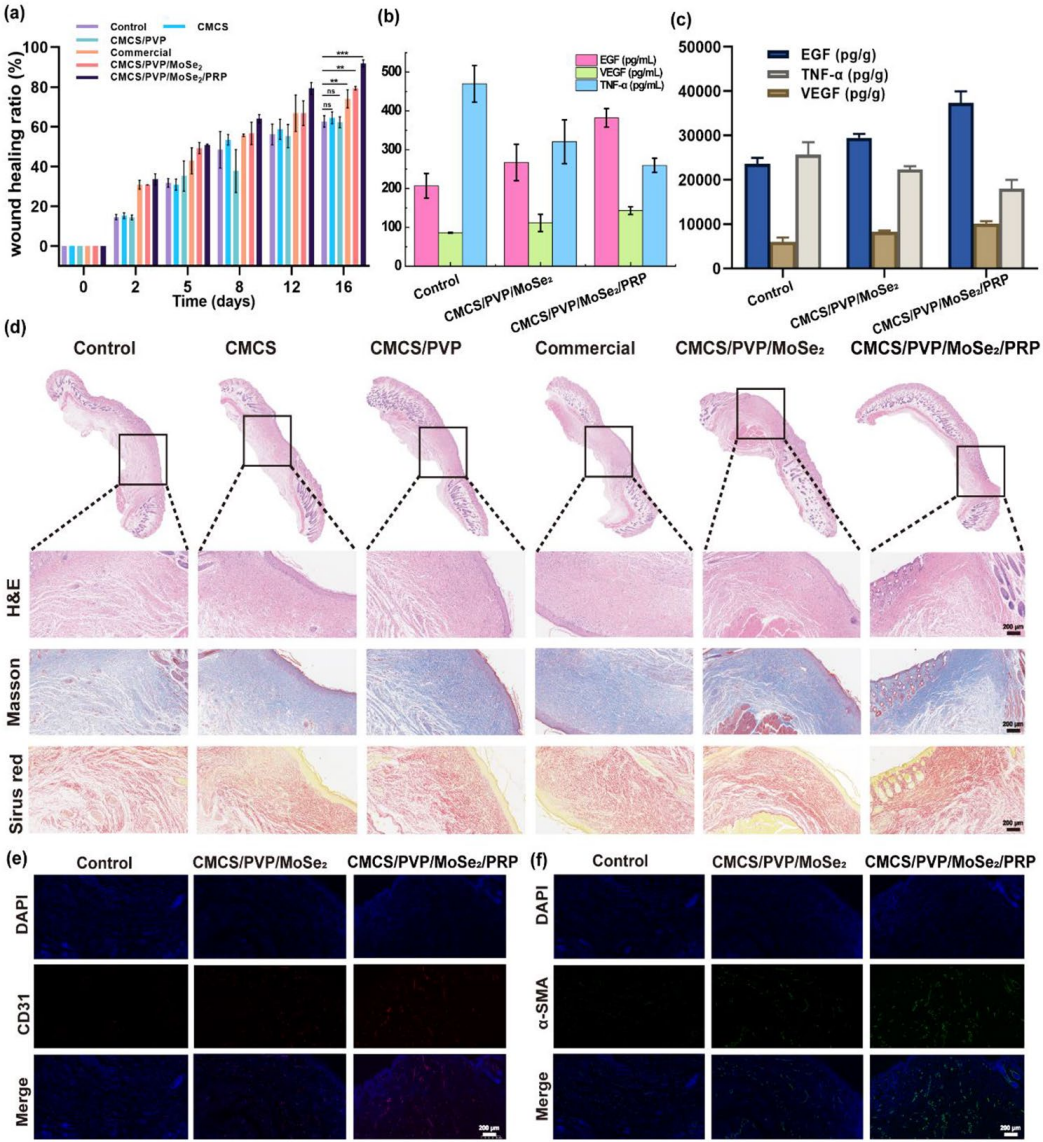
**(a)** Wound healing ratio in Balb/c mice treated with different hydrogels on days 0, 2, 5, 8, 12, and 16; **(b)** levels of EGF, VEGF, and TNF-α in serum of mice treated with different hydrogels (day 16); **(c)** levels of EGF, VEGF, and TNF-α in mouse tissues treated with different hydrogels (day 8 and day 16). **(d)** representative images of H&E staining, Masson staining, and Sirius Red staining of the healing skin of Balb/c mice obtained on day 16 (Bar = 200 μm); **(e)** representative images of CD31 staining of healing skin of Balb/c mice obtained on day 16 (Bar = 200 μm); **(f)** representative images of α-SMA staining of healing skin of Balb/c mice obtained on day 16 (Bar = 200 μm)

Wound infection is one of the leading causes of death in injured patients, and prolonged inflammation leads to excessive free radical production at the wound site, further leading to tissue damage and delayed wound healing. Having explored that CMCS/PVP/MoSe_2_/PRP hydrogels exhibit good cell proliferation properties in fibroblast cell lines, we wanted to explore their therapeutic efficiency in animal models. The wound healing promotion was checked by recording digital photographs of the wound area over 16 days. The results showed that the wound area decreased over time in both the control and hydrogels groups (Fig. 7e). Compared with the control, CMCS, CMCS/PVP and commercial group, the unclosed wound areas in the CMCS/PVP/MoSe_2_ hydrogels group and CMCS/PVP/MoSe_2_/PRP hydrogels group were smaller at different postoperative time points. In addition, the area was significantly reduced in the CMCS/PVP/MoSe_2_/PRP hydrogels group compared to the CMCS/PVP/MoSe_2_ hydrogels group (Fig. 7e). After 16 days of treatment, the wounds in the CMCS/PVP/MoSe_2_/PRP hydrogels group were almost completely closed, with a healing ratio of 90.63 ± 0.48%, i.e., the CMCS/PVP/MoSe_2_/PRP hydrogels showed better wound healing than the other groups (Fig. 8a). This wound healing promotion was attributed to the combined antioxidant, cell proliferation, and anti-inflammatory effects of CMCS/PVP/MoSe_2_/PRP hydrogels.

Then, the expression of the inflammatory factor TNF-α and the growth factors EGF and VEGF were detected in mouse serum (day 16) as well as in skin tissues (day 8 and day 16) using an ELISA assay. The CMCS/PVP/MoSe_2_/PRP hydrogel group showed the highest expression of EGF and VEGF and the lowest expression of TNF-α in both serum and skin tissues. This suggests that the hydrogel effectively reduced the inflammatory response compared with the other groups, and the increase in the levels of EGF and VEGF suggests that the cells effectively proliferated and migrated to promote wound healing (Fig. 8b-c and S3). Further, we analyzed the collected tissue samples for pathological staining. H&E and Masson staining suggested that all groups had newly formed epidermis and increased granulation tissue thickness, and the CMCS/PVP/MoSe_2_/PRP hydrogels group was superior to the other groups (Fig. 8d). Collagen formation at the wound site is crucial in re-modelling damaged skin tissue and enhancing stretch. As shown in Fig. 8d, Sirius red staining indicated that collagen deposition was more pronounced in the CMCS/PVP/MoSe_2_/PRP hydrogels group, further indicating that CMCS/PVP/MoSe_2_/PRP hydrogels could promote collagen fiber growth and accelerate the wound healing. Angiogenesis is necessary for tissue remodelling and was studied by CD31 staining and α-SMA staining. CD31 is a transmembrane protein expressed in early angiogenesis, indicating neovascularisation. α-SMA is a cytoplasmic protein that is highly expressed in late angiogenesis, indicating maturation of vascular smooth muscle cells. As shown in Fig. 8e-f and S4a-b, the relative fluorescence intensity images and quantitative results of CD31 and α-SMA antibodies indicate that the dermal vascular density of wounds healed with CMCS/PVP/MoSe_2_/PRP was significantly higher compared to control, CMCS hydrogel, CMCS/PVP hydrogel, commercial and CMCS/PVP/MoSe_2_ hydrogel-treated groups. In summary, these results above suggest that due to the introduction of MoSe_2_ and PRP, CMCS/PVP/MoSe_2_/PRP hydrogels synergistically scavenge free radicals, anti-inflammatory and release growth factors and contribute to angiogenesis, which ultimately significantly improves wound healing.

## Conclusions

In conclusion, we successfully designed a multifunctional CMCS/PVP/MoSe_2_/PRP hydrogels with antioxidant and growth factor-releasing functions to treat skin injury and promote wound healing. In the preparation process, we introduced MoSe_2_ and PRP into the hydrogels by a simple physical mixing method. The prepared multifunctional hydrogels have good mechanical properties, swelling capacity, and antioxidant properties. Moreover, the three-dimensional structure of the hydrogels endowed the hydrogels with a sustained growth factors release capability from PRP. Therefore, the CMCS/PVP/MoSe_2_/PRP hydrogels not only scavenged ·OH, O_2_·^−^ and DPPH free radicals but also maintained the bioactivity of the PRP, which promoted cell proliferation and migration. In a mouse model, the hydrogels were validated to promote tissue re-epithelialization, collagen deposition, and accelerated angiogenesis with significant wound healing effects. In conclusion, this study provides a highly informative solution for treating skin injuries and promoting wound healing.

### Electronic supplementary material

Below is the link to the electronic supplementary material.

Supplementary Material 1

## Data Availability

The data that support the findings of this study are available from the corresponding author upon reasonable request.
