# Supplementary material for: Molybdesum selenide-based platelet-rich plasma containing carboxymethyl chitosan/polyvinyl pyrrolidone composite antioxidant hydrogels dressing promotes the wound healing
Source: J Nanobiotechnology. 2024 May 9;22:217. doi: 10.1186/s12951-024-02490-9 (PMC11080249; doi:10.1186/s12951-024-02490-9)
Supplement: Supplementary file 1 — Supplementary Material 1 [file 12951_2024_2490_MOESM1_ESM.docx]

**Supplementary Information**

**Molybdesum selenide-based platelet-rich plasma containing carboxymethyl chitosan/polyvinyl pyrrolidone composite antioxidant hydrogels dressing promotes the wound healing**

Xiaoyi Zheng,^a,1^ Yongliang Ouyang, ^b,1^ Hengwei Fan,^c,1^ Liying Zhang,^b^ Shige Wang,^b^ Yanbo Zeng,^a, *^ Lianghao Hu,^a, *^ and Jiulong Zhao ^a,*^

^a^ Department of Gastroenterology, Changhai Hospital, Naval Medical University, No. 168 Changhai Road, Shanghai 200433, P. R. China

^b^ School of Materials and Chemistry, University of Shanghai for Science and Technology, No. 516 Jungong Road, Shanghai 200093, P. R. China

^c^ Department of Hepatic Surgery Department, the Eastern Hepatobiliary Surgery Hospital, Navy Medical University, No. 225 Changhai Road, Shanghai 200438, P. R. China

# **Supplementary content**

- 1. **Experimental section**

**1.1.1 In vitro antimicrobial assay**

We validated the antibacterial activity of hydrogels in liquid media, using Escherichia coli (*E. coli*) (Gram-negative) and Staphylococcus aureus (*S. aureus*) (Gram-positive) as bacterial models to study the antibacterial activity of hydrogels. All bacteria were cultured in Luria-Bertani broth (LB) medium, with a group of pure LB medium without bacterial liquid as a blank group and a group of LB medium tubes with bacterial liquid as a control group. The remaining experimental groups were CMCS, CMCS/PVP, Commercial (human epidermal growth factor), CMCS/PVP/MoSe_2_ and CMCS/PVP/MoSe_2_/PRP groups, respectively, where hydrogels (1 g) were co-cultured with the medium. All subgroups were in a 15 mL glass test-tube containing 1.5 mL medium and 1.5 mL bacterial/medium solution. The seeded bacterial density was determined by reading the bacterial/medium solution absorbance at 625 nm to be ~ 0.15. There tubes were incubated at 37 ◦ C in an incubator for 1 d. Three parallel experiments were set up for each group. Finally, the bacterial suspensions from the different treatments were collected and their absorbance was measured at 625 nm. The bacteriostatic ratio was calculated from Eq x**.**

$Bacteriostatic ratio \left( \% \right)=\frac{A_{p}-A_{i}}{A_{p}}\times100\%$ (x)

where A_p_ is the light absorbance of the control liquid medium at 625 nm and A_i_ is the light absorbance of the liquid medium in the experimental group at 625 nm. Then, we verified the bacteriostatic properties of CMCS/PVP/MoSe_2_/PRP hydrogels by using the inhibition zone method in solid medium. Resuscitated *E. coli* and *S. aureus* were added to LB solid medium. Pre-prepared disc-shaped CMCS; CMCS/PVP; Commercial; CMCS/PVP/MoSe_2_ and CMCS/PVP/MoSe_2_/PRP hydrogels (Thickness: 2 mm) were attached to the surface of the LB medium. Finally, the petri dishes were incubated in a constant temperature incubator at 37°C for 24 h to observe the effect of the inhibition zones.

**2.1 Results and discussion**

**2.1.1 In vitro anti-bacterial capacity evaluation**

Bacterial infection may have a profound effect on wound healing. Therefore, we tested whether the hydrogel has antimicrobial effect. It was found that the inhibition rates of all hydrogels were lower than 7% for *E. coli* and lower than 6% for *S. aureus* (Figs. S1a, b). Moreover, the inhibition zones of all the five hydrogels were not significant, indicating that our hydrogels do not have significant bacteriostatic properties (Figs. S1c, d). Therefore, to obtain an admirable antimicrobial effect, antibiotics need to load into the hydrogel in future.

# **Supplementary figures**


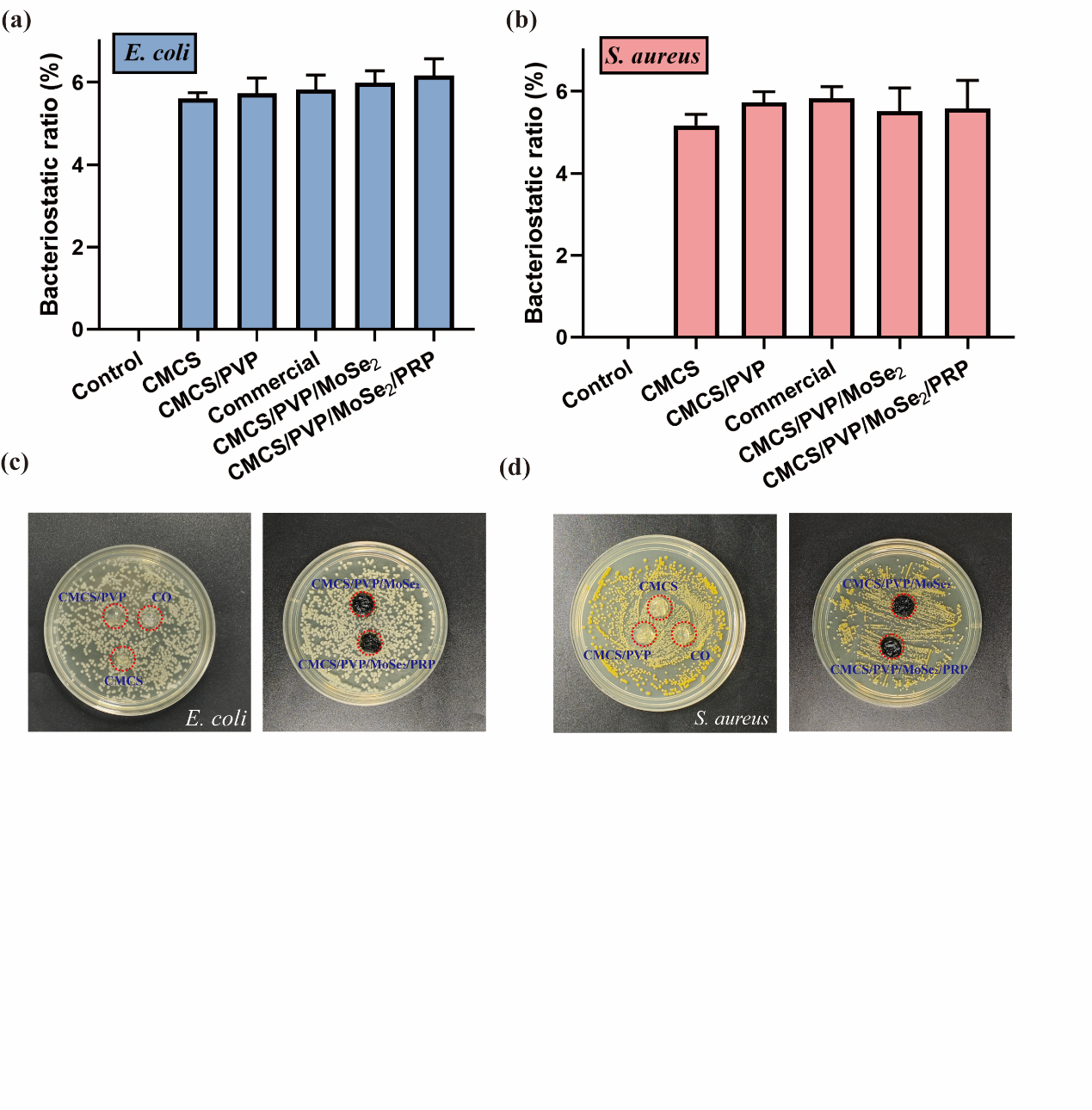


**Fig.S1** a) Bacteriostatic ratio of *E. coli* by different hydrogels; b) Bacteriostatic ratio of *S. aureus* by different hydrogels; c) photographs of the *E. coli* inhibition zones (CO is human epidermal growth factor: commercial); d) photographs of the *S. aureus* inhibition zones.


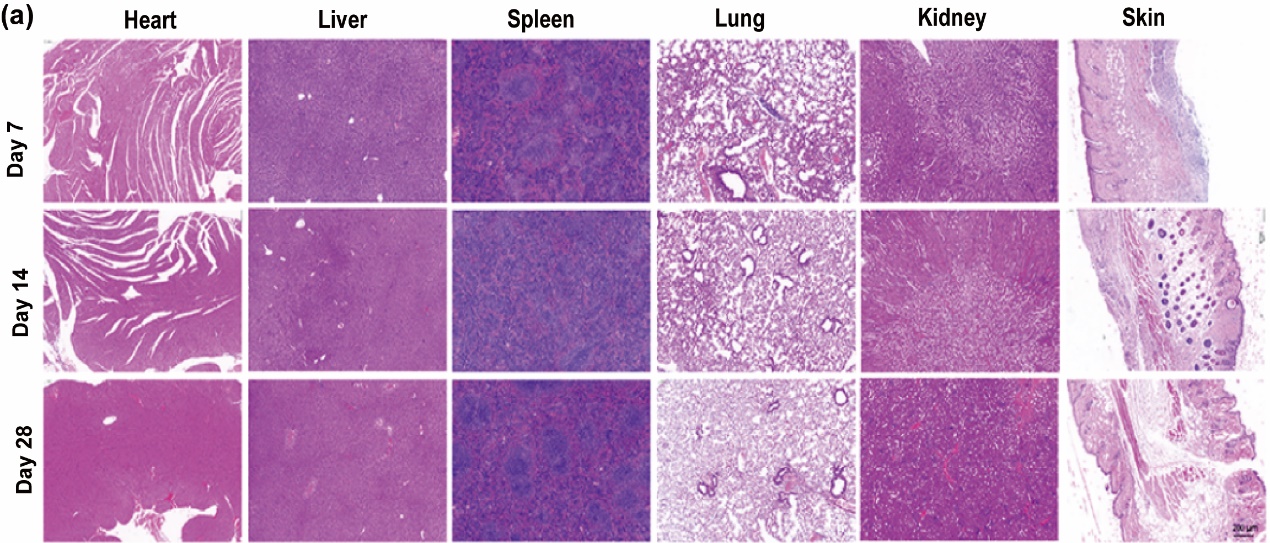


**Fig.S2** a) H&E staining images of major organs and skin at hydrogels embedding sites of hydrogels treated KM mice.


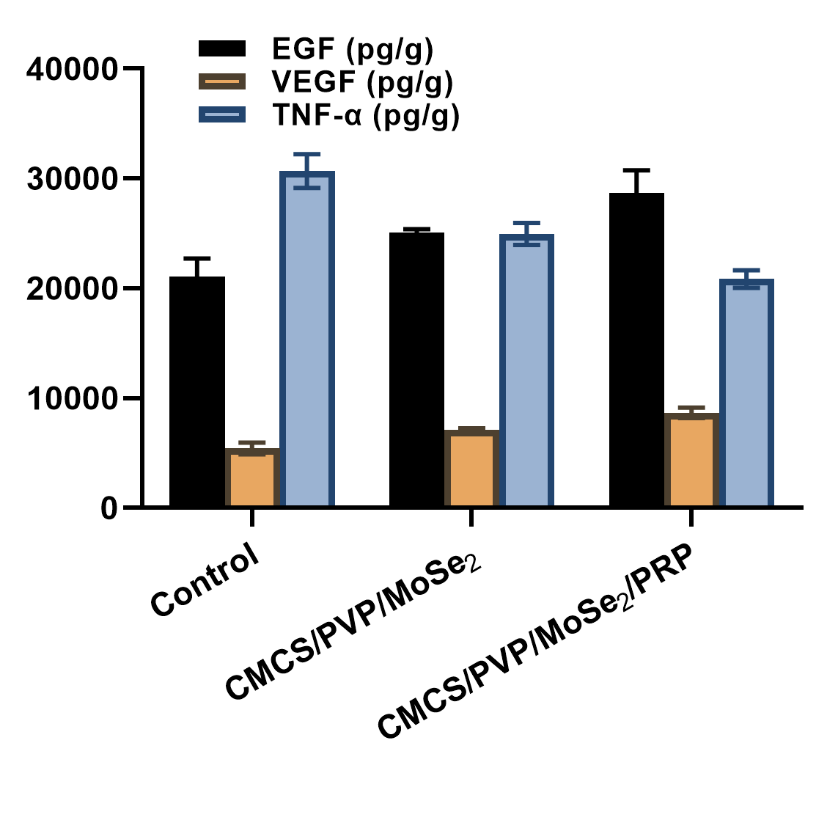


**Fig.S3** Levels of EGF, VEGF and TNF-α in mouse tissues treated with different hydrogels (day8)


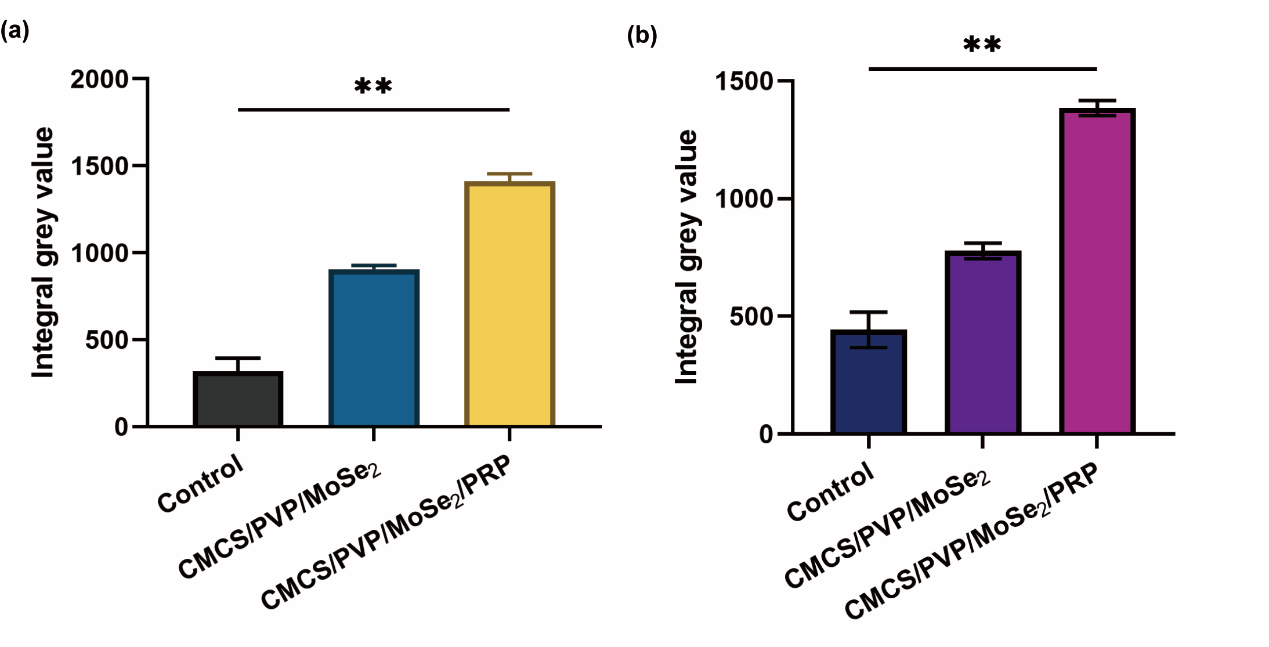


**Fig.S4** a) Fluorescence integral grey value of CD31; b) fluorescence integral grey value of α-SMA.
